# First-line therapy of peripheral T-cell lymphoma: extension and long-term follow-up of a study investigating the role of autologous stem cell transplantation

**DOI:** 10.1038/bcj.2016.63

**Published:** 2016-07-29

**Authors:** M Wilhelm, M Smetak, P Reimer, E Geissinger, T Ruediger, B Metzner, N Schmitz, A Engert, K Schaefer-Eckart, J Birkmann

**Affiliations:** 1 Hematology and Medical Oncology Clinic, Paracelsus Medical University Nuernberg, Nürnberg, Germany; 2 Evang. Krankenhaus Essen-Werden, Clinic for Hematology, Medical Oncology and Stem Cell Transplantation, Pattbergstr, Essen, Germany; 3 Institute of Pathology, University of Wuerzburg, Comprehensive Cancer Center Mainfranken, Würzburg, Germany; 4 Institute of Pathology, Karlsruhe, Germany; 5 University Clinic for Internal Medicine - Hematology and Oncology Clinic, Klinikum Oldenburg, Oldenburg, Germany; 6 Asklepios Hospital St. Georg, Hematology Department, Hamburg, Germany; 7 University Hospital of Cologne, Internal Medicine I, Köln, Germany

## Abstract

Current guidelines recommend consolidation with autologous stem cell transplantation (autoSCT) after induction chemotherapy for most patients with peripheral T-cell lymphoma (PTCL). This assumption is based on five prospective phase II studies, three of which included <50 patients with limited follow-up. Here we present the final analysis of the prospective German study. The treatment regimen consisted of four to six cycles of CHOP chemotherapy followed by mobilizing therapy and stem cell collection. Patients in complete remission (CR) or partial remission (PR) underwent myeloablative chemo(radio)therapy and autoSCT. From January 2001 to July 2010, 111 patients were enrolled in the study. The main subgroups were PTCL not specified (*n=*42) and angioimmunoblastic T-cell lymphoma (*n=*37). Seventy-five (68%) of the 111 patients received transplantation. The main reason for not receiving autoSCT was progressive disease. In an intent-to-treat analysis, the complete response rate after myeloablative therapy was 59%. The estimated 5-year overall survival, disease-free survival and progression-free survival rates were 44%, 54% and 39%, respectively. The results of this study confirm that upfront autoSCT can result in long-term remissions in patients with all major subtypes of PTCL and therefore should be part of first-line therapy whenever possible.

## Introduction

Peripheral T-cell lymphomas (PTCL) comprise a group of rare and heterogeneous hematologic malignancies, characterized by an aggressive disease course chronology in order.^1^ Despite the fact that T-cell lymphomas have a poor outcome after conventional chemotherapy, the optimal therapy for these lymphomas remains to be determined.^2, 3^ One reason for this is that most studies on the treatment of T-cell lymphomas are difficult to interpret owing to retrospective analysis, inclusion of subgroups with a better prognosis (that is, ALK-positive anaplastic large cell lymphoma (ALCL)), and small patient numbers.^4, 5, 6, 7, 8, 9, 10^ In addition, owing to the rarity of the disease, randomized prospective studies are currently not available. Therefore, for defining a treatment standard, we must preferentially rely on prospective phase II studies. Owing to the dismal prognosis of patients with PTCL treated with conventional chemotherapy, to date, four other prospective studies specifically dedicated to PTCL investigated the role of upfront autologous stem cell transplantation (autoSCT).^11, 12, 13, 14, 15^

In July 2006, Corradini *et al.*^13^ published the combined results of two prospective phase II studies investigating the role of intensified sequential chemotherapy followed by upfront autoSCT in patients with PTCL with estimated 12-year overall survival (OS), disease-free survival (DFS) and event-free survival (EFS) rates of 34%, 55% and 30%, respectively. These results have to be interpreted with caution, as 19 of the total 62 patients (31%) had ALK-positive ALCL, a subentity with a much better prognosis than all other PTCL entities. The analysis of the remaining 43 patients with PTCL yields an OS of 21% and an EFS estimate of 18%.

In the small study published by Rodriguez *et al.*^15^ in 2007, 19 of 26 patients received autoSCT, with 3-year OS and progression-free survival (PFS) rates of 85% and 59%, retrospectively.

A Spanish group reported a 4-year OS rate of 39% in 41 patients with PTCL in 2008. In this study only 41% of the 41 patients eventually received autoSCT.^14^

These three studies have a relatively small sample size if the good prognosis patients are excluded from analysis and therefore, the possibility of substantial bias needs to be considered.

In January 2009, a paper by our group presented data on patients with PTCL who were treated with upfront myeloablative radiochemotherapy and autoSCT following four to six cycles of conventional chemotherapy.^11^ For this prospective study, 83 patients were enrolled, excluding ALK-positive ALCL as well as PCTL. At the time of publication, the estimated 3-year OS, PFS and DFS rates were 48%, 36% and 53%, respectively.

In July 2012, d'Amore *et al.*^12^ published the results of a large trial of the Nordic Lymphoma Group (NLG-T-01). In this study, 160 patients with PTCL, also excluding ALK-positive ALCL, were treated with conventional chemotherapy followed by consolidation with high-dose chemotherapy and autoSCT. The 5-year estimates of OS and PFS were 51% and 44%, respectively.

This study was first published in 2009 when 83 patients had been included. Additional 28 patients were treated thereafter according to the study protocol. Here we present the analysis of the in total 111 patients with a median follow-up of almost 5 years. Our study is still the largest German study and the second largest worldwide with a long follow-up.

## Patients and methods

### Patient characteristics

After this study was reported in 2009 we decided to treat all consecutive patients according to the study protocol.^11^ Thus, until July 2010, additional 28 patients were treated exactly as stipulated by the study protocol. The outcome of these patients is reported together with a follow-up report on the original cohort of the 83 patients reported previously.

Altogether between January 2001 and July 2010, 111 patients with newly diagnosed PTCL were treated. Inclusion criteria were as follows: histologically confirmed PTCL, histology reviewed by the Reference Center for Lymph Node Pathology in Wuerzburg, Germany, age 18–65 years, ECOG performance status <4, no severe comorbidity, no pregnancy or lactation, and written informed consent. The study protocol did not allow to include patients with primary cutaneous lymphomas and ALCL.

The median age was 49 years (range 23–66 years). The vast majority of the patients presented with advanced disease: 83 patients (75%) had stage III–IV disease. The age-adjusted international prognostic index was low/intermediate low in 44 patients (40%) and intermediate high/high in 64 patients (58%). Patient characteristics are listed in Table 1.

### Treatment plan

The treatment plan has been described before.^11^ In brief, patients received four cycles of CHOP chemotherapy. If no complete remission (CR) was achieved, two more cycles were allowed. Patients in CR and patients achieving at least a partial remission (PR) after six cycles proceeded to mobilization chemotherapy.

For mobilization of hematopoietic stem cells, the DexaBEAM protocol (dexamethasone, carmustine, etoposide, cytarabine and melphalan) was used in 71 of the 84 patients. For 13 patients the ESHAP protocol (etoposide, methylprednisolone, cytarabine, cisplatin) was administered. A minimum harvest of 5 × 10^6^ CD34^+^ cells was required for the patient to be eligible for high-dose therapy.

Myeloablative radiochemotherapy consisted of fractionated TBI 2 × 2 Gy/d on days –6 to –4 for a total dose of 12 Gy, followed by high-dose cyclophosphamide (60 mg/kg body weight) on days –3 and –2 in 54 patients. As published studies suggest that TBI is not mandatory to give optimal results in T NHL patients, the more recent patients (*n=*21) received high-dose therapy according to the BEAM protocol (carmustine 300 mg/m^2^ day –7, etoposide 200 mg/m^2^ day –6 to day –3, cytarabine 2 × 200 mg/m^2^ also day –6 to day –3, melphalan 140 mg/m^2^ day –2). On day 0 autologous stem cells were transfused.

### Response criteria

All lesions clinically and/or radiologically involved were measured bidimensionally by the respective investigator. Treatment response was assessed as CR, PR, stable disease or progressive disease according to the Cheson criteria.^16^ Response was evaluated after four cycles of CHOP therapy, before myeloablative therapy and when clinical findings suggest relapse or progression of disease. After the autoSCT, response was assessed at 3-month intervals during the first two years and at 6-month intervals thereafter.

### Statistical methods

All patients entering the study were evaluated on an intention-to-treat basis. OS was calculated from the date of diagnosis to the date of death from any cause. PFS was defined as the period from the date of initiation of treatment to the date of relapse, disease progression or lost to follow-up. DFS for patients who achieved a CR was calculated from the date of the first documentation of a CR to the date of the first relapse. OS, PFS and DFS rates were estimated using the Kaplan–Meier method. The median follow-up time was calculated as proposed by Schemper and Smith.^17^ Data were run through the Kaplan–Meier analysis using deaths and censoring reversed. To identify prognostic variables for OS, univariate analysis was performed.

## Results

### Treatment response

**Figure 1:**
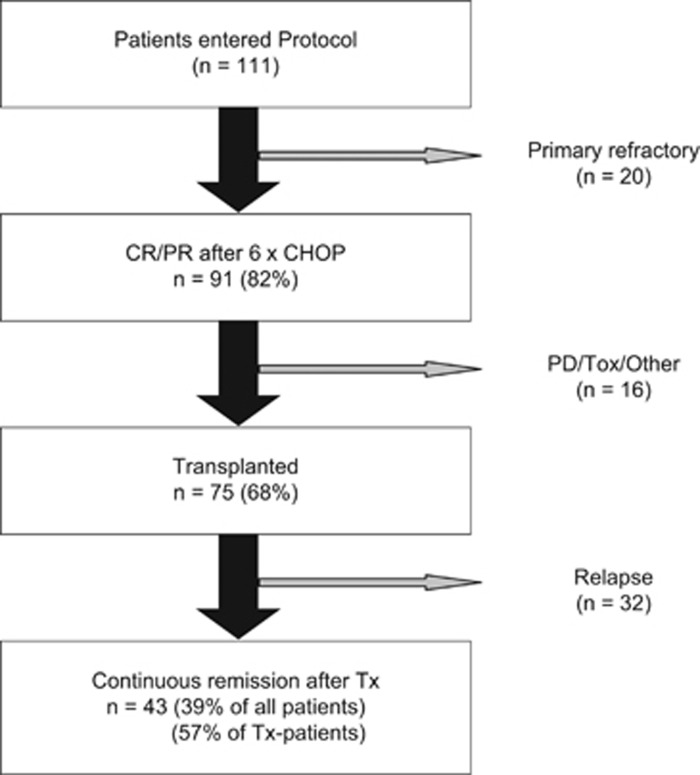
Course of Treatment of the Study Patients (*n=*111). CHOP: cyclophosphamide, doxorubicin, vincristine and prednisone; CR: complete remission; PR: partial remission; Tx: stem cell transplantation; PD: progressive disease; Tox: toxicity.

The intention-to-treat population comprised 111 patients with PTCL eligible for high-dose chemotherapy. Of these 111 patients, 91 (82%) responded to the initial CHOP therapy. Sixty-nine of them (62%) achieved a CR, 22 patients (20%) a PR. Twenty patients (18%) failed to achieve a remission and thus were treated off study. The course of treatment of the 111 patients is summarized in Figure 1 and Table 2.

Of the 91 responders to initial chemotherapy, 84 patients started stem cell mobilizing therapy. Seven patients were not mobilized because of early relapse or withdrawal of consent. Despite successful mobilization of a sufficient number of stem cells using either method, only 75 of the 84 patients undergoing mobilization therapy proceeded to high-dose therapy and autoSCT. The reasons for not undergoing autoSCT were disease progression after initial response (*n=*5), fatal toxicity (*n=*2) and withdrawal of consent (*n=*2). Thus, two-thirds (68%) of the intention-to-treat population completed the entire study protocol.

The majority of patients (*n=*54) received myeloablative radiochemotherapy with 12 Gy total body irradiation followed by 120 mg/kg cyclophosphamide, the remaining 21 patients received high-dose chemotherapy according to the BEAM protocol. Before high-dose therapy and autoSCT, 63 of these 75 patients (84%) were in CR and 12 patients (16%) in PR; after the autoSCT procedure two more patients, that is, 65 patients (87%), achieved a CR.

### Follow-up and survival

Of those 75 patients who completed the whole study treatment, 32 relapsed after autoSCT. With respect to the intention-to-treat population of 111 patients, 39% achieved a continuous remission. The estimated 5-year OS, DFS and PFS were 44%, 54% and 39%, respectively.

**Figure 2:**
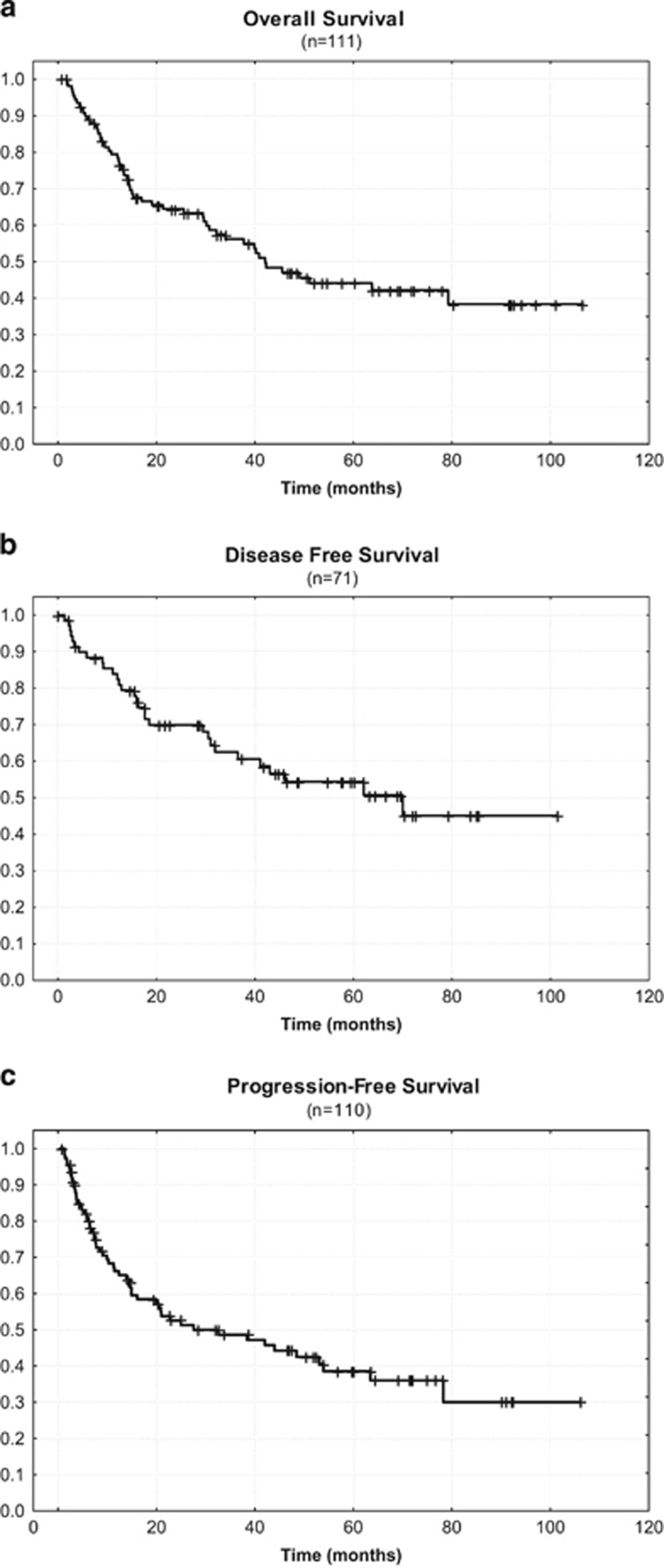
Kaplan–Meier curves for (**a**) overall survival, (**b**) disease-free survival and (**c**) progression-free survival.

**Figure 3:**
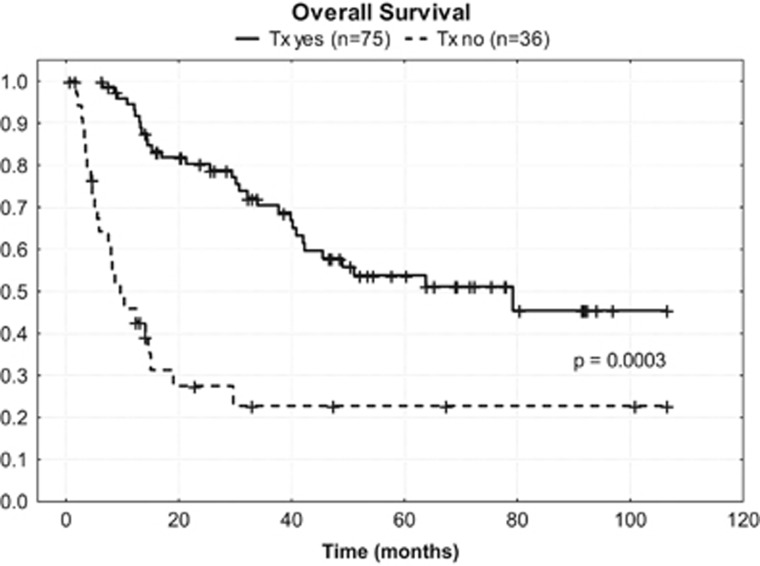
Kaplan–Meier curves for overall survival in patients who did and did not receive transplantation.

At a median follow-up time of 59 months (range 1 to 107 months), 58 patients (52%) were still alive, and 43 of them (39%) were in continuous remission. In addition, 15 of them (14%) were alive with disease. Figure 2 show the Kaplan–Meyer plots with respect to OS (A), DFS (B) and PFS (C). The estimated 5-year OS rate was 57% for patients who underwent autoSCT compared with 23% for patients who did not undergo SCT (Figure 3; *P=*0.0003).

### Toxicity

Treatment-related morbidity according to the WHO criteria in this study was comparable with other high-dose studies and did not significantly differ from the earlier reported initial evaluation. Overall four patients died from treatment-related infectious complications, resulting in a TRM rate of 3.6%.

### Prognostic parameters

**Figure 4:**
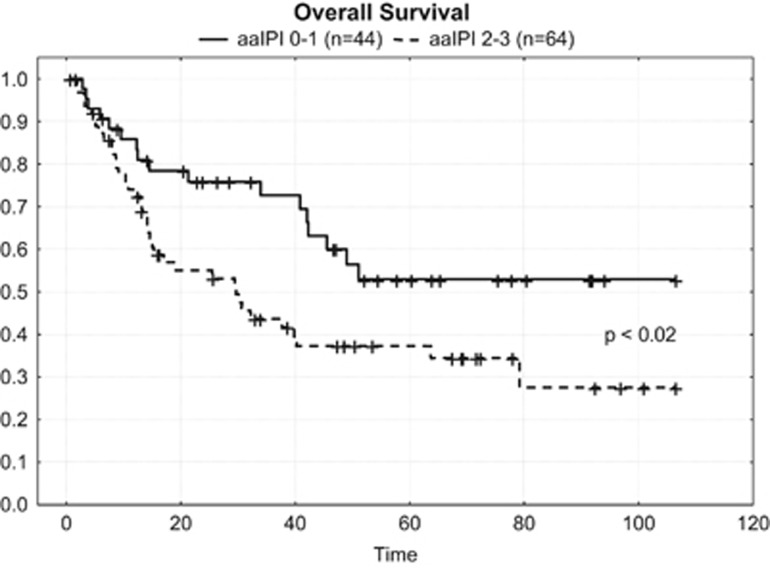
Kaplan–Meier curves for overall survival according to aaIPI of the patients.

In contrast to the results of the International T-cell Lymphoma Project, univariate analysis regarding the impact of histologic subtype on OS did not reveal a significant correlation as was the case regarding age, stage, B symptoms and elevated lactate dehydrogenase. This might be caused by the fact that the proportion of patients with limited disease and without the mentioned risk factors was relatively small. In contrast, univariate but not multivariate analyses revealed that there was a significant correlation between age-adjusted international prognostic index low/low intermediate vs high/high-intermediate and longer OS (Figure 4; *P<*0,02).

## Discussion

PTCL represent an aggressive disease with a mostly dismal prognosis after conventional chemotherapy.^1, 5, 7^ Also owing to their low incidence and their considerable heterogeneity, a standard therapy has not yet been defined.^18, 19^ With regard to intensive chemotherapy followed by upfront autoSCT, five phase II studies have been reported. (Tables 2 and (1)). Although the studies published by Rodriguez *et al.* (*n=*14), Mercadal *et al.* (*n=*41) and Corradini *et al.* (*n=*43) are hampered by relatively small sample size, our own study first published in 2009 had a relatively short follow-up of 33 months.^11, 13, 14, 15^ The trial recently reported by d'Amore *et al.*^12^comprised a relatively large number of patients (*n=*160), whereas the prospective phase II trial published by Corradini *et al.* had the longest follow-up of 76 months.^13^ Taken together, we believe that all studies added substantial evidence that intensive chemotherapy followed by autoSCT is a valid strategy of first-line therapy in patients with PTCL. However, we still felt it difficult to define upfront autoSCT as standard therapy in PTCL and therefore decided to extend the observation time and treat more patients according to the protocol of our previous study. Here we present the final analysis of 111 patients treated, with a median follow-up of 59 months.

Several aspects of our study are important when comparing the results with the other trials investigating the role auf autoSCT for evaluation of treatment standards. All five PTCL trials are prospective phase II trials (Table 2) avoiding a bias of analyzing only chemosensitive patients and patients with potentially more risk factors (Tables 1 and 2). The vast majority of the patients in each trial presented with advanced stage disease (from 75 to 96%). Further risk factors, such as B symptoms, elevated serum lactate dehydrogenase levels or an age-adjusted international prognostic index of high-intermediate or high, were predominant in all of the study populations discussed here.^6^

Before inclusion in our study, reference pathology was mandatory as it is known that the accurate diagnosis of PTCL can be challenging with a relatively low likelihood of a concordant final diagnosis at a referring institution.^20^ Central revision also was an inclusion criterion in the studies by d'Amore, Mercadal and Rodriguez.

The International T-cell Lymphoma Project has shown differences in survival according to histology, with 5-year OS ranges from 90% for PCTL to 7% for hepatosplenic PTCL.^1, 4^ Therefore, entities with the best prognosis after conventional chemotherapy (ALK-positive ALCL and PCTL) usually have not been included in trials investigating the role of autoSCT as part of first-line therapy except that published by Corradini *et al.* (in which an additional analysis excluding ALK-positive ALCL was made). However, with exception of ALK-positive ALCL in the Corradini trial and ALK-negative ALCL in the d'Amore trial, subgroup analysis in the other studies including our own (data not shown) did not reveal significant outcome differences according to the most frequent subentities. This discrepancy might be explained by the low number of patients in the relevant subgroups compared with the International T-cell Lymphoma Project or the ‘equalizing' effect of autoSCT.

Conventional chemotherapy consisted of four to six cycles of the CHOP regimen in all patients in our trial, whereas d'Amore *et al.* used the CHOEP-14 regimen in patients age <60 years.^12^ Corradini *et al.*, Mercadal *et al.* and Rodriguez *et al.* used different induction chemotherapies.^13, 14, 15^ The debate about the best induction therapy will continue, as neither the more aggressive drug combinations nor the CHOP protocol, with or without etoposide, were able to prevent early progression in 16–41% of the PTCL patients. The retrospective analysis published by Schmitz *et al.* on 343 patients treated within trials of the German High-Grade Non-Hodgkin Lymphoma Study Group (DSHNHL) showed an improvement of the 3-year event-free survival when etoposide had been added to the CHOP protocol, but preferentially in the ALK-positive ALCL group and in patients younger than 60 years and with normal lactate dehydrogenase levels, that is, in a more favorable subgroup.^21^ Similar results for younger patients have been reported by Ellin *et al.*^3^ Likewise, the role of monoclonal antibodies like alemtuzumab remains unclear, as any additional effect on response is hampered by considerable toxicity, especially life-threatening opportunistic infections.^22^ Taken together, although CHOP may not be the ideal protocol for inducing remission in PTCL, a truly better regimen has not yet been defined.

There is also a lack of standards regarding the optimal mobilization therapy and/or high-dose protocol before transplantation. Although all reported mobilization therapies proved effective in yielding a sufficient stem cell harvest, their value for increasing CR rates is questionable. The same holds true for the different high-dose chemotherapy and/or radiochemotherapy protocols, which only moderately contributed to the augmentation of CR rates, but obviously are of utmost importance for achieving long-term remissions (Table 2).

This update of the trial published in 2009, reporting more patients with a longer follow-up, confirms the early study results. The survival curves did not much deteriorate over time and the survival plateaus indicate that long-term survival can be achieved after high-dose therapy/ autoSCT in a substantial fraction of patients able to proceed to it. Only few deaths occurred beyond 50 months post transplant (Figure 2a). An intention-to-treat analysis of our study at a median follow-up time of 59 months reveals that 43 of the 111 patients (39%) achieved a continuous remission and 58 patients (52%) are still alive, with or without disease. Despite the fact that different first-line chemotherapies applied slightly differ, the strategy of using a dose-dense conventional chemotherapy followed by upfront high-dose chemotherapy with autologous stem cell rescue is very similar in all prospective trials. In the intention-to-treat patient population, a long-term OS of >40% and a PFS of >30% can be achieved (Table 2). A major problem remains that mainly due to failure of induction therapy and early relapse, only about two-thirds of the intention-to-treat population will be able to proceed to transplantation. In each trial, patients who completed the whole program showed significantly better OS compared with those who failed early (Figure 3).

However, it is difficult to identify at diagnosis those patients who will have the greatest benefit from upfront autoSCT. In an univariate analysis, IPI defines risk groups in Corradini, d̀Amore, and Mercadal̀s trials, as well as in our patient group. Nevertheless, different risk groups according to IPI should not alter the therapeutic strategy, as even IPI low patients have a disappointing treatment outcome.

Is allogeneic stem cell transplantation (alloSCT) able to improve results?^23, 24, 25, 26, 27^ Corradini *et al.*^28^ conducted a prospective trial using reduced-intensity conditioning. Owing to the high response rate and low transplantation-related mortality of this trial, alloSCT was considered a promising approach in PTCL for younger patients. In the meantime, a prospective, randomized, European multicenter trial has been performed (‘AATT trial') to determine the role of upfront alloSCT in PTCL. Unfortunately, this study was stopped prematurely after an interim analysis had shown that the primary end point, that is, improvement of EFS by alloSCT, could not be met with the patient numbers planned to be enrolled.^29^ Thus, at present, there is no evidence that alloSCT is superior to CHO(E)P chemotherapy plus upfront autoSCT in first-line therapy of most PTCL. However, for extremely aggressive subtypes, such as hepatosplenic lymphoma or adult T-cell leukemia/lymphoma (ATLL), upfront alloSCT might be an option, as graft-versus-leukemia activity can result in long-term survival in a significant proportion of patients.^30, 31, 32^

The major challenge for the future is to increase response rates and bring more patients to transplantation. Many new drugs are being investigated. Brentuximab vedotin and crizotinib appear to be promising agents in subentities of PTCL.^33, 34^ Romidepsin, belinostat and pralatrexate yield remarkable responses in relapsed patients.^35, 36, 37, 38, 39^ Once the phase III trials with these drugs are finished, we will know whether these or other new drugs will improve response rates, allowing more patients to achieve the possibility of undergoing autoSCT.^40^

In conclusion, based on available data, upfront autoSCT should be recommended as a rational choice for patients with the nodal subtypes PTCL-NOS, AITL, ALK-negative ALCL, as well as high-risk ALK-positive ALCL eligible for high-dose chemotherapy.^5^ However, PFS remains disappointing ranging from 30 to 50%. Nevertheless, this treatment strategy likely improves the outcome for PTCL patients compared with conventional chemotherapy.

## Figures and Tables

**Table 1:** Clinical characteristics of patients at diagnosis

	**n**	***%***
Overall	111	100
NOS	42	38
AITL	37	33
ALK-negative ALCL	16	14
Intestinal	7	6
Lennert-lymphoma	1	1
NK/T, nasal-type	5	5
Hepatosplenic	3	3
Median age (years)	49	
range	23–66	
Sex ratio (male/female)	67:44	60:40
		
*Stage (Ann Arbor)*		
I or II	28	25
III or IV	83	75
B symptoms	64	58
		
*AaIPI score*		
Low/low intermediate	44	40
High/high-Intermediate	64	58
Unknown	3	3
Elevated LDH	64	58

Abbreviations: AaIPI, age-adjusted International Prognostic Index; AITL, angioimmunoblastic T-cell lymphoma; ALCL, anaplastic ALK-negative large cell lymphoma; LDH, lactate dehydrogenase; NOS, not otherwise specified; PTCL, peripheral T-cell lymphoma.

**Table 2:** Prospective studies on high-dose therapy and autotransplantation in PTCL as first-line therapy

*Author*	*n*	*Age*	*Regimen*	*Response before Tx*	*Tx-rate*	*OS*	*Follow-up*
Present study	111	49	Cy/TBI or BEAM	62% CR 20% PR 18% PD	68%	44% (5-year)	59
Mercadal^14^	41	47	BEAM/BEAC	49% CR 10% PR 39% PD	41%	39% (4-year)	38
D́Amore^12^	160	57	BEAM/BEAC	51% CR 30% PR 16% PD	72%	51% (5-year)	61
Corradini^13^^a^	62	43	Mito/Mel or BEAM	56% CR 16% PR 24% PD	74%	34/21%^b^ (12-year)	76
Rodriguez^15^	26/14^c^	44	BEAM	65% CR 12% PR 19% PD	73%	73% (3-year)	35

Abbreviations: CR, complete remission; PD, progressive disease; PR, partial remission; OS, overall survival.

a Combined analysis of two separate studies, which included ALK-positive ALCL.

b OS for all patients and non-ALK-positive histology, respectively.

c Only 14/26 patients in this study received autoSCT as upfront therapy. Analysis includes all patients.
